# Detection of Microsatellite Instability by High-Resolution Melting Analysis in Colorectal Cancer

**DOI:** 10.52547/ibj.26.1.70

**Published:** 2021-12-20

**Authors:** Nafiseh Raji, Tayebeh Majidi Zadeh, Pegah Babheidarian, Massoud Houshmand

**Affiliations:** 1 Pajouhesh Blvd., Karaj Highway, Department of Medical Genetics, National Institute of Genetic Engineering and Biotechnology, Karaj, Iran;; 2 Shahid Hemmat Highway, Iran University of Medical Sciences, Tehran, Iran

**Keywords:** Biomarkers, Microsatellite instability, Colorectal cancer

## Abstract

Background:

Colorectal cancer is the third most common cancer worldwide. MSI is a molecular marker of a deficient MMR system and happens in almost 15% of CRCs. Because of a wide frequency of MSI^+^ CRC in Iran compared to other parts of the world, the importance of screening for this type of cancer is highlighted.

Methods:

The most common MSI detection technique is a fluorescent PCR-based method in which fragments are analyzed by CE. This technique is very time-consuming, difficult, and expensive. We sought to develop and evaluate a proper method with high accuracy, specificity, and sensitivity to screen the MSI^+ ^CRC. A HRM analysis procedure is relying on the analysis of the melting curve attributes. Low cost, feasibility, high specificity, and sensitivity are outstanding attributes of HRM analysis.

Results:

Five mononucleotide microsatellite markers, including BAT-25, BAT-26, NR-21, NR-24, and NR-27, in 25 archival CRC tumor tissue samples were compared with normal tissue adjacent using HRM method. The specificity and sensitivity of BAT-25 with HRM method were 100% compared to CE, while other markers had lower sensitivity. However, when all the markers were considered together, the sensitivity and specificity became 100%. The number of MSI^+^ samples was 56%, which shows a higher ratio than previous Iranian studies. The highest MSI was related to BAT-26 (52%).

Conclusion:

The HRM method is much simpler and more cost-effective than current MSI techniques, and its sensitivity and accuracy are comparable. Therefore, it can serve as an alternative method in cases where CE is unavailable.

## INTRODUCTION

Colorectal cancer is the third most prevalent cancer worldwide and the third prominent cause of cancer-related deaths in Iran^[^^1^^]^. There is an approximately 5% risk of CRC in the general population. The risk rate of this cancer in Iran is less than that of Western countries, but similar to other regions of the Middle East. Moreover, the proportion of CRC in young population is significantly higher in Iran than Western societies. Studies have demonstrated that about 5-10% of CRC is due to genetic background; however, this rate is about 20% in Iran^[^^2^^]^. 

Some of the tumorgenesis pathways in CRC include chromosomal instability, MSI, and epigenetic factors^[^^3^^]^. MSI is a kind of genomic instability due to inefficient repair of insertion/deletion of repetitive units during DNA replication^[^^4^^,^^5^^]^. Indeed, MSI is a prognostic marker in almost 15%–20% of sporadic CRC. It is also a firmly established screening marker for Lynch syndrome (2–5% of all CRCs). The MMR deficiency is caused by mutations in one of the MMR genes (*MLH1*,* MSH2*,* MSH6*, and *PMS2*). Past and prospective evidence has suggested that cells with defects in MMR proteins are resistant to 5-FU. Also, patients with Lynch syndrome are also resistant to 5-FU^[^^6^^]^. PCR-based MSI detection in CRC is traditionally based on a format of five microsatellite markers (two mononucleotide repeats and three dinucleotide repeats) offered by the National Cancer Institute Research Workshop in Bethesda, Maryland^[^^7^^]^. While some laboratories use different panels of markers, the majority use the NCI panel or a commercially available kit from Promega (USA). Recent studies have shown that mononucleotide markers have specificity and sensitivity higher than dinucleotide markers for detecting positive MSI. However, Promega uses mononucleotide markers BAT-25, BAT-26, NR-21, NR-24, and NR-27^[^^6^^,^^7^^]^_._ Due to importance of CRC diagnosis and the high percentage of MSI^+^ in Iran relative to other regions of the world, screening for MSI^+^ is an urgent need^[^^8^^,^^9^^]^. 

The goal of this study was to use an appropriate technique with high accuracy and sensitivity for screening MSI^+^ CRC. Current MSI techniques include fluorescent PCR-based experiments, in which the fragments are analyzed, or DNA is sequenced by denaturing HPLC^[^^5^^]^. However, these methods have significant limitations; MSI analysis with the usual standard approach is relatively expensive, difficult, and time-consuming. There is an alternative method, HRM, which uses the closed-tube post-PCR. HRM employs highly saturated double-stranded DNA dyes and is a potentially helpful method for the evaluation of sequence diversity^[^^10^^]^. Using HRM, samples can be identified based on sequence length, GC content and complementary DNA sequences. HRM can also be utilized in genotyping applications, such as insertion/deletion analysis of single nucleotide polymorphisms and unknown genetic mutations^[^^5^^,^^10^^]^. Therefore, the HRM method can display heteroduplexes formed in MSI^+^ tumors because of alteration in the length (indel) of microsatellites. Moreover, the formation and analysis of stutter peaks can be avoided by applying this method^[^^5^^]^. 

Herein, we evaluated HRM analysis for five mononucleotide microsatellite markers, i.e. BAT-25, BAT-26, NR-24, NR-21, and NR-24, in NR-27 archival CRC samples. 

## MATERIALS AND METHODS

**Sample collection and DNA preparation**

Archived-FFPE samples from 25 Iranian patients, which were histologically confirmed as CRC, were acquired from the Pathology Department of Rasoul Akram and Imam Khomeini Hospitals, Tehran, Iran. Areas enriched in cancer cells were highlighted by a pathologist and applied for tumor DNA extraction. MSI-positive control DNA samples were obtained from Tehran University of Medical Sciences. These controls were instable for all five markers (BAT-25, BAT-26, NR-21, NR-24, and NR-27). DNA from 25 cancerous tissues and 25 normal adjacent tissues were extracted by phenol chloroform. The quantity and the quality of extracted DNA samples were checked with a Nanodrop 2000 spectrophoto-meter (Thermo Fisher Scientific, USA).

**HRM analysis**

The primer sets for BAT-25, BAT-26, NR-21, NR-24, and NR-27 mononucleotide marker panel were designed as described before^[^^11^^]^. Primer sequences of the mentioned markers are represented in Table 1. Using Corbett Rotor-Gene 6000 real-time PCR Cycler (Qiagen), HRM protocol was set as follows: initial denaturation at 95 °C for 12 min and then 50 cycles of denaturation at 95 °C for 10 s, annealing at 55 °C (for all five genes) for 20 s, and elongation at 72 °C for 10 s. The final step included the melting curve analysis (0.2 °C step increments, 2 or 4 s hold before each acquisition) from 65 to 95 °C. The PCR amplification and HRM were performed in a final volume of 20 μL containing 4 μl of 5× Hot FirePol^®^ EvaGreen^®^_,_ 10 pmol*/*μl of each primer, and 10 ng*/*μl of DNA. All experiments were repeated at least three times for evaluating the reproducibility of the results. Rotor-Gene 6000 Software (version 1.7.87) was used for the analysis of melting curves obtained on the Corbett instrument. In the Genotype section of the Rotor-Gene 6000 Software program (version 1.7.87), we assigned positive control as MSI. Results could be viewed as either a normalized melt plot or a difference plot. Normalized curves provided the basic representation of different genotypes based on curve shifting and curve shape change. Difference in the data points within the regions were used to normalize fluorescence (the Y-axis only) for the start (Region 1) and end (Region 2) of the melting plot. Data outside the set regions were ignored. The regions were adjusted to encompass representative baseline data for the pre-melt and post-melt phases. Difference plots provide an alternative view of the differences between melt curve transitions. We used determined reference curves to subtract normalized and temperature shifted curves to produce different curves, which were automatically collected into segregate groups. Samples with two curves specified for various groups were recorded as MSI^+^, and samples with at least one reference curve as MSS. Using the mononucleotide marker panel, a sample was defined as MSI^+^ when it showed instability with at least two or more markers, and MSS when it showed no instability for markers or instability with only one marker. To confirm the MSI assay by HRM method, some samples were analyzed by CE. MSI^+^ samples and MSS stable samples were obtained. We calculated the analytical sensitivity considering the number of true-positive samples divided by the total number of true-positive and false-negative samples, multiplied by 100. We also defined analytical specificity considering the number of true-negative samples divided by the total number of true-negative and FP samples, multiplied by 100. ROC curves were utilized to measure the speciﬁcity and sensitivity in anticipating the MSI based on the HRM. The area under the ROC curve and 95% conﬁdence intervals were computed. 

**Table 1:** Primer sequences for MSI assay

**MSI markers**	**Primer sequences**	**Amplicon size** ** (bp)**
BAT-25-F	TCGCCTCCAAGAATGTAAGT	120
BAT-25-R	TCTGCATTTTAACTATGGCTC	
		
BAT-26-F	TGACTACTTTTGACTTCAGCC	130
BAT-26-R	AACCATTCAACATTTTTAACCC	
		
NR-21-F	GAGTCGCTGGCACAGTTCTA	109
NR-21-R	CTGGTCACTCGCGTTTACAA	
		
NR-24-F	GCTGAATTTTACCTCCTGAC	131
NR-24-R	ATTGTGCCATTGCATTCCAA	
		
NR-27-F	AACCATGCTTGCAAACCACT	87
NR-27-R	CGATAATACTAGCAATGACC	

F, forward; R, reverse

**Statistical analysis**

For the statistical analysis, the MSI phenotypes were divided into two groups: the MSS and MSS were assigned as “0”, and the MSI^+^ group as “1”. Age was continuous variable, while gender, differentiation, and stage were categorical variables. Statistical analysis was performed using the IBM SPSS statistics 24 and the Graphpad prism 8 software. The correlation between two variables was evaluated using Pearson’s χ^2^, and logistic regression analysis was conducted to rank the factors associated with MSI and identify the predictors of MSI. Statistical significance was defined as *p* < 0.05. Multivariate analysis for distribution categorical variables indicated by asymptotic χ^2 ^and computing OR (95% CI).

**Ethical statement**

The above-mentioned sampling protocols were approved by the Research Ethics Committee of National Institute of Genetic Engineering and Biotechnology, Tehran, Iran (ethical code: IR. NIGEB.EC.1399.4.9.B). Written informed consents were provided by all the patients. 

## RESULTS

**Fig. 1:**
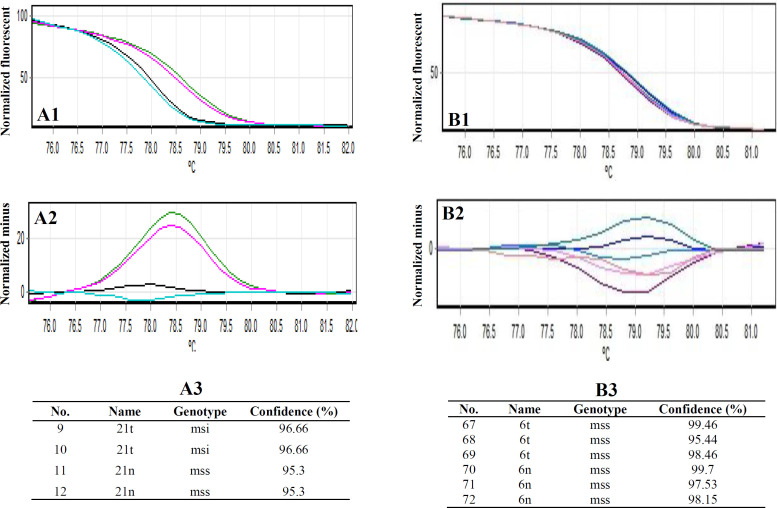
**HRM result. HRM-shifted melting and difference curves result for BAT-26 (A1,** **A** **2,** **A3)** **and NR-21** **(** **B1,** **B** **2,** **B** **3) markers. (A)** **S** **ample 21 was obtained with two repetitions in one run for BAT-26 unstable; (B) sample 6 was obtained with three replications in one run for NR-21 as normal (MSS)**

A total of 25 sporadic CRC patients (12 females and 13 males) with the median age of 55.5 years (ranged between 39 and 72) were participated in the study. The HRM technique was used to examine five mononucleotide markers, i.e. BAT-25, BAT-26, NR-21, NR-24, and NR-27, in a total of 25 CRC samples. Samples with both curves were considered as MSI^+^, and samples with at least one curve, as a reference, were regarded as MSS (Fig. 1).

Instability was observed in the tumoral DNA compared to the normal adjacent tissue DNA sample. For BAT-25 both sensitivity and specificity of the HRM technique was 100%, the sensitivity and specificity for BAT-26, NR-24, NR-27 were 73.33 and 100%, but 73.33 and 86.66% for NR-21, respectively. By using the mononucleotide marker panel, we detected MSI in 14 out of 25 cases (56%) and 11 MSS (44%) with CRC. The most instable marker, BAT-26, was detected in 13 cases (52%). Among 14 MSI-H patients, instability of BAT-25 occurred in 11 cases (44 %), NR-27 in 10 cases (40%), NR-21 in 9 cases, (36%) and NR-24 in 6 cases, (24%). Moreover, instability of two markers was recognized in 36%, three markers in 4%, four markers in 12%, and all the markers in 8% of the patients. Based on the results, the rate of MSI-H in males was greater than females (36% vs. 24%), and the difference was not statistically significant (*p* = 0.072). Clinicopathological characteristics of the study population are presented in Table 2.

## DISCUSSION

CRC is divided into three categories. The majority of CRCs develop through the chromosome instability pathway, while 12–15% arises from the MSI pathway, owing to defect in MMR. MMR deficiency can be caused by an inherited genetic mutation in one of the MMR genes (*MLH1*, *MSH2*, *MSH6*, and *PMS2*) or more due to the epigenetic inactivation of MLH1 gene and CpG island methylation phenotype^[^^12^^]^. The role of MSI as a genetic marker for Lynch syndrome is well known. MSI and IHC testing are effective methods for determining tumors caused by MMR deficiency and are highly sensitive methods for identifying a defective MMR system. guiding physicians to select most valuable and cost-effective genetic testing. 

MSI testing is positive but IHC study is negative, when there is an inactive protein^[^^13^^]^. MMR defects occur due to a genetic mutation in one of the MMR genes, followed by a second hit on that gene allele or methylation of the MMR gene promoter (often MLH1), which results in the loss of protein function^[^^14^^]^. Studies have displayed that MSI-H tumors possess phenotypic aspects such as poor differentiation, proximal colon location, and abundance of tumor-infiltrating lymphocytes^[^^12^^]^. 

Unlike MSS, MSI-H is predictive for medical treatment outcome in chemotherapy and immuno-therapy and associated with desirable results, including more desirable prognosis, a higher five-year survival, and lower metastatic potential^[^^11^^]^. Among the early stage CRCs with MSI-H, stage II is associated with higher survival rate compared to stage III patients. In patients with stage II colon cancer and MSI-H, there is no benefit of 5-FU adjuvant-based chemotherapy^[^^12^^]^. Despite the similarity of the sickness stages, CRC patients showsignificant differences in clinical outcome, possibly due to molecular heterogeneity of the tumor. Thus, the molecular classification of CRC can detect patient subtypes at different risk of recurrence and death and may be useful for personalizing treatment modalities^[^^11^^,^^12^^]^. There is evidence that CRCs have intrinsic instability and if adequate markers are examined, most of the CRCs have some degrees of MSI^[^^15^^]^, showing a continuing phenotype, as represented recently by massively parallel sequencing^[^^11^^]^. A range of other cancers, such as endometrial, intestinal and gastric cancers, also have MSI phenotypes, indicating a defect in the mismatch repair system and the prominent role of MSI beyond colon cancer^[^^16^^]^. Inaccurate assessment of MSI can lead to the loss of patients who would otherwise benefit from immune therapy and likely have a strong and durable tumor response. Treatment with an immune checkpoint inhibitor has more efficiency in patients with MSI-H colon cancer that has been accentuated by mismatch repair deficiency^[^^17^^]^. Therefore, the diagnosis of MSI has a great clinical importance. 

**Table 2:** Clinicopathological characteristics of the patients

**Characteristics**	**MSI (n)**	**MSS (n)**	**Odds ratios**	**95% CI**
Gender				
Female	6	6	1.291 (0.1686-12.15)	-1.780 to 2.497
Male	8	5		
				
Stage				
II	12	1	0.2149 (0.02907-0.9878)	-3.538 to -0.01227
III	1	7		
IV	1	2		
				
Differentiation				
Well	4	7	0.5914 (0.1229 to 2.491)	-2.096 to 0.9128
Moderately	11	3		

Some techniques are deed based on ﬂuorescent-labeled sequencing analysis (CE) and DHPLC for MSI analysis. However, these methods have significant limitations as they are relatively time-consuming, difficult, and expensive. DHPLC is based on hetero- or homo-duplex formation and melting temperature-related immigration of annealed amplicons via a solid-phase column. DHPLC has benefits over the CE-based methods because it is devoid of the stutter peaks intrinsic to CE, therefore giving appropriate exegesis^[^^5^^]^.

HRM point analysis is a technique based on DNA melting technique. The behavior of HRM separation in DNA samples is characterized by their change from double stranded DNA to single stranded DNA that occurs with increasing temperature. HRM is comparable to DHPLC in its analytical specificity and sensitivity. The major disadvantages of the DHPLC method are chemical waste, high preservation expense, the requirement for post-PCR manipulations, and down throughput^[^^18^^]^. Another significant downside of DHPLC is that due to several amplicon melting temperatures, the column temperature needs to be optimized for each target to achieve the optimal point of denaturation. Unlike DHPLC, which depends on the specific temperature in order to be strongly optimized for each run, HRM analysis scans a wide range of temperatures. Despite screening multi-gene panels to detect single-point mutations, microsatellites are more likely to harbor indels because of a lack of mismatch repair. Hence, MSI screening in small sets of predefined markers may be more effective and less costly way for use in clinical classification. As recently suggested for point mutations, such as KRAS, multiplexed HRM may be employed to prescreen the presence of MSI-positive samples, whereas negative HRM samples may need further investigation and can be classified as stable MSI^[^^19^^]^. HRM analysis with DNA saturated dyes, as a scanning tool for heteroduplex detection, has obvious benefits, including ease of assay design, no PCR operation, precision, versatility, high analysis speed, and cost efficiency^[^^18^^]^.

In the Dietmaier *et al.*’s^[^^20^^]^ study, the ability of real-time PCR (hybridization probe) was investigated to detect MSI^+^ samples. The results showed the capability of this technique to detect MSI^+^ samples up to one deletion nucleotide in the microsatellite sequence. In the study of Mokarram *et al.*^[^^8^^]^, blood serum samples were compared for non-invasive screening, and their microsatellite status was compared with normal and tumor tissue samples using real-time PCR and HPLC techniques. Their results suggested that the real-time PCR technique has the same sensitivity and specificity as the reference method for detecting MSI^+^, whereas HPLC was less sensitive and specific. In addition, blood serum was not a good sample for screening MSI^+^ tumors^[^^8^^]^.

One of the great advantages of HRM over other genotype techniques, e.g. sequencing and Taqman single nucleotide polymorphism typing, is its cost-effectiveness. This exceptional feature makes it ideal for large scale genotyping projects. Speed is other advantage of HRM, which can genotype a large number of samples in a short time. The simplicity of the method is also a prominent feature of HRM analysis as an attractive tool for genotyping and application in diagnostic laboratories. For these reasons, we applied HRM technique for MSI typing of a total of 25 sporadic CRC patients. In this study, MSI phenotype was diagnosed in 56% of patients, which is higher than the previous reports^[^^1^^,^^8^^,^^9^^]^. The highest MSI was related to BAT-26 in 13 cases (52%).

The frequency of MSI in sporadic CRC was found to be 15-20%. There is a higher percentage of MSI^+^ in Iran than anywhere else in the world^[^^8^^,^^9^^[^. In Esmailnia's^]^^1^^[^ study, the MSI was detected in 36 out of 80 cases (45%) with CRC. Bishehsari and colleagues^[^^21^^]^ observed that the percentage of sporadic tumors of MSI^+^ was 4.19%; however, its prevalence was 26% in the Moghbeli *et al.*’s study^[^^9^^]^ and 9.26% in the Brim *et al.*’s study^[^^22^^]^. In the study of Zeinalian and co-workers^[^^23^^]^ conducted on patients from Isfahan, seven cases of BAT-26, six cases of BAT-25 and NR-24, and five cases of NR-21 and NR-27 were found with MSI. There are many reports of instability among the five mononucleotide markers in other Iranian studies that are very different and contradictory. Our results were confirmed by fragment analyses with CE. We also acquired identical results when we carried out MSI typing for six MSI samples by fragment analyses for five mononucleotide repeats (BAT-25, BAT-26, NR-21, NR-24, and NR-27). Therefore, the reference method was exploited in parallel with this method. 

**Fig. 2:**
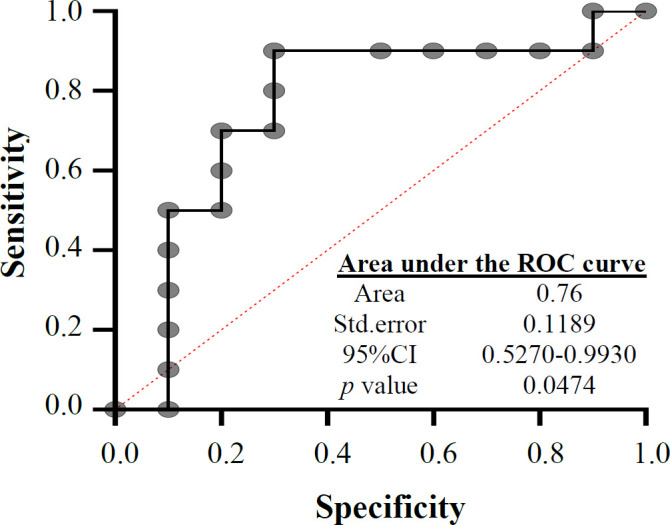
ROC curve for MSI

As the positive samples with CE had at least two unstable markers by the HRM method, the sensitivity of MSI^+^ samples were 100%. The sensitivity and specificity of BAT-25 were 100% using HRM method in comparison with CE. Sensitivity of BAT-26, NR-21, NR-24, and NR-27 was lower (73.33). The specificity of all of these markers was 100% except for NR-21, which was 86.66. To evaluate the specificity of this method, we examined three samples that had previously been detected as MSS by fragment analysis using the HRM method. We observed no FP for the BAT-26, NR-24, NR-25, and BAT-27 markers, i.e., an analytical specificity of 100%. A FP sample was observed for the NR-21 marker (analytical specificity of 86.66%). However, with the evaluation of all the five markers and the existence of instability in more than 40% (two out of five) of markers, the sensitivity and specificity became 100%.

ROC analysis was used to determine the accuracy and sensitivity of the identification method in separating the microsatellite stability and instability of tumor and normal samples. The results indicated a level below the chart, implying the predictive power of MSI marker. Predictive power above 70% exhibits the appropriateness of the measurement method. The sensitivity and speciﬁcity of HRM for MSI markers with ROC curve were 76% (Fig. 2), which indicates that the HRM can be used as a suitable diagnostic method for further studies. 

Herein, we observed that most of the MSI^+^ samples were from stage II, and the MSS samples were from stage III/IV (Table 2). In future study, MSI should be evaluated in the early stages, such as stage I, in order to take the necessary preventive measures at the earliest time. Overall, we recommend doing HRM as a method with high sensitivity once fragment analysis is not accessible. Moreover, HRM can serve as an appropriate method for screening MSI tumors in CRC samples.

## CONFLICT OF INTEREST.

None declared.
